# Quality of life 1 month after acute pulmonary embolism in emergency department patients

**DOI:** 10.1111/acem.14692

**Published:** 2023-03-08

**Authors:** Anthony J. Weekes, Jillian Davison, Kathryn Lupez, Jaron D. Raper, Alyssa M. Thomas, Carly A. Cox, Dasia Esener, Jeremy S. Boyd, Jason T. Nomura, Kathleen Murphy, Patrick M. Ockerse, Stephen Leech, Jakea Johnson, Eric Abrams, Christopher Kelly, Nathaniel S. O’Connell

**Affiliations:** 1 Department of Emergency Medicine, Atrium Health’s Carolinas Medical Center, Charlotte, North Carolina, USA; 2 Department of Emergency Medicine, Orlando Health, Orlando, Florida, USA; 3 Department of Emergency Medicine, Kaiser Permanente, San Diego, California, USA; 4 Department of Emergency Medicine, Vanderbilt University Medical Center, Nashville, Tennessee, USA; 5 Department of Emergency Medicine, Christiana Care, Newark, Delaware, USA; 6 Department of Emergency Medicine, University of Utah Health, Salt Lake City, Utah, USA; 7 Department of Biostatistics and Data Science, Wake Forest University School of Medicine, Winston-Salem, North Carolina, USA; 8 Department of Emergency Medicine, Tufts Medical Center, Boston, Massachusetts, USA; 9 Department of Emergency Medicine, University of Alabama at Birmingham, Birmingham, Alabama, USA; 10 Emergency Department, Houston Methodist Baytown Hospital, Houston, Texas, USA; 11 Emergency Medicine of Idaho, Meridian, Idaho, USA

## Abstract

**Objective:**

The Pulmonary Embolism Quality-of-Life (PEmb-QoL) questionnaire assesses quality of life (QoL) after pulmonary embolism (PE). We aimed to determine whether any clinical or pathophysiologic features of PE were associated with worse PEmb-QoL scores 1 month after PE.

**Methods:**

In this prospective multicenter registry, we conducted PEmb-QoL questionnaires. We determined differences in QoL domain scores for four primary variables: clinical deterioration (death, cardiac arrest, respiratory failure, hypotension requiring fluid bolus, catecholamine support, or new dysrhythmia), right ventricular dysfunction (RVD), PE risk stratification, and subsequent rehospitalization. For overall QoL score, we fit a multivariable regression model that included these four primary variables as independent variables.

**Results:**

Of 788 PE patients participating in QoL assessments, 156 (19.8%) had a clinical deterioration event, 236 (30.7%) had RVD of which 38 (16.1%) had escalated interventions. For those without and with clinical deterioration, social limitations had mean (±SD) scores of 2.07 (±1.27) and 2.36 (±1.47), respectively (*p* = 0.027). For intensity of complaints, mean (±SD) scores for patients without RVD (4.32 ± 2.69) were significantly higher than for those with RVD with or without reperfusion interventions (3.82 ± 1.81 and 3.83 ± 2.11, respectively; *p* = 0.043). There were no domain score differences between PE risk stratification groups. All domain scores were worse for patients with rehospitalization versus without. By multivariable analysis, worse total PEmb-QoL scores with effect sizes were subsequent rehospitalization 11.29 (6.68–15.89), chronic obstructive pulmonary disease (COPD) 8.17 (3.91–12.43), and longer index hospital length of stay 0.06 (0.03–0.08).

**Conclusions:**

Acute clinical deterioration, RVD, and PE severity were not predictors of QoL at 1 month post-PE. Independent predictors of worsened QoL were rehospitalization, COPD, and index hospital length of stay.

## INTRODUCTION

Pulmonary embolism (PE) is the third most common cardiovascular disease and it has an annual incidence of 0.6 to 1.0 per 1000 in Europe and the United States.^1,2^ When a patient presents to the emergency department (ED) or to their primary care physician with a PE, the physician ideally will use a validated risk stratification tool to determine how best to treat the patient based on disease severity. Most risk stratification strategies, however, do not consider early quality of life (QoL; within weeks of patient disposition) as an outcome.^3,4^ Functional limitations are common after venous thromboembolism (VTE) and likely impair QoL.^5–7^ The literature, however, is limited on patient QoL shortly after a PE diagnosis.

Although concerns about PE severity and acute clinical deterioration influence risk stratification, it is unclear if early clinical deterioration impacts subsequent QoL. The presence of abnormal right ventricular (RV) physiology is an important marker of the acute physiologic cardiac burden of PE. The persistence of RV abnormalities and development of chronic thromboembolic pulmonary hypertension can be associated with decreased cardiopulmonary fitness. However, this complication is uncommon. It is reported in just 2%–4% of those with a previous PE.^8^ Cardiopulmonary fitness may also be impacted, though, by the treatment delivered. In one study, 35% of intermediate-risk PE patients treated with anticoagulation monotherapy had elevated RV pressure measurements at 6 months versus 7% of those treated with anticoagulation and systemic thrombolysis.^9^ The anticoagulation monotherapy cohort had worse 6-month cardiopulmonary fitness compared to the anticoagulation and thrombolysis cohort.

In this study, our primary objective was to determine if any clinical or pathophysiologic features of PE detected during the index PE presentation were associated with patient QoL 1 month post-PE. We used the validated Pulmonary Embolism Quality-of-Life (PEmb-QoL) questionnaire to measure this primary outcome. PEmb-QoL was developed and validated in 2009.^10^ The validation study was conducted on patients who had PE diagnosed between 2001 and 2007 and completed the PEmb-QoL between 10 and 91 months after the index PE diagnosis.^11^ Our secondary objective was to determine if there was a difference in domain QoL at 1 month between groups of PE patients with an abnormal RV who did or did not receive escalated PE interventions.

## METHODS

### Study design and setting

This prospective, observational, multicenter study is a planned analysis of two registry databases that were previously reported.^12^ The first database was the Pulmonary Embolism Short-term Clinical Outcomes Registry (PESCOR; clinicaltrials.gov NCT02883491). Patients in this first database were enrolled between August 2016 to March 2019. The second registry, distinguished only by federal funding (clinicaltrials.gov NCT03915925: Short-term Clinical Deterioration After Acute Pulmonary Embolism), enrolled patients between September 2018 and December 2020. Both registries were populated by the same sites and had similar variables, data recording instruments, and outcome variables. The six enrolling sites for both registries were academic EDs in San Diego, California; Newark, Delaware; Orlando, Florida; Charlotte, North Carolina; Nashville, Tennessee; and Salt Lake City, Utah.

The study was approved by each institution’s review board. Initially, informed consent was required for telephone contact. Later the central site’s institutional review board (IRB) approved a waiver of written informed consent and allowed patients to be contacted for voluntary participation in the PEmb-QoL survey. With federal funding, a single IRB approved the protocol with waiver of informed consent.

### Study population

Each site enrolled men and women 18 years or older, who had acute PE diagnosed within 12 h of ED presentation and were willing to complete the PEmb-QoL questionnaire 1 month later. To be included in the study, the PE had to be confirmed by (1) presence of filling defects in pulmonary arteries on contrast-enhanced computed axial tomography of the chest, (2) high-probability findings on ventilation perfusion nuclear imaging, or (3) detection of thrombus in the heart or pulmonary artery by transthoracic echocardiography or transesophageal echocardiography. Patients who declined any participation or were under police custody during the study were excluded.

### Data collection to populate registries

The clinical course of enrolled patients was monitored for 5 days and up to 30 days after index PE. The following data were abstracted from electronic medical records: patient demographics, prehospital events, vital signs, comorbidities, Charlson comorbidity index, PE risk factors, other clinical information relevant to calculating PE risk stratification scores, and length of hospital stay. Patients were monitored during the hospitalization for predefined clinical deterioration endpoints (defined below). For patients discharged directly from the ED or discharged within 2 days of admission to the hospital, the electronic medical record was reviewed for clinical status or subsequent rehospitalization within sites’ affiliated networks during the 30 ± 3 days after PE. Data used for reporting or statistical analysis were recorded in a REDCap database.^13^

### Measurements

We studied four primary independent variables: (1) acute clinical deterioration, (2) right ventricular dysfunction (RVD), (3) initial PE risk stratification, and (4) subsequent rehospitalization. Below we define data collection specific to each variable in the registry databases analyzed for this study. As in previous studies, acute clinical deterioration was defined as a composite of death or other predefined adverse events occurring within 5 days of PE diagnosis in the ED.^12,14,15^ The adverse events included cardiac arrest, respiratory failure, hypotension requiring fluid bolus or catecholamine support, and new dysrhythmia.

RVD was determined based on point-of-care goal-directed echocardiography (GDE) performed during the index PE ED evaluation by emergency medicine (EM) attendings credentialed in point-of-care ultrasound and EM residents in training. GDE digital video images of the parasternal long- and short-axis apical four-chamber and subcostal four-chamber views were archived. All six study sites had academic EM residency programs with advanced emergency ultrasound fellowship programs directed by the site investigator for this study. As independent adjudicators, site investigators interpreted the archived GDE images using guidelines for RV assessments in PE.^16–18^ Prior reports have demonstrated the accuracy of our GDE in PE approach compared to comprehensive echocardiography, sharing both high inter- and high intra-rater agreement.^16^

Severe RV dilation was determined by an absolute RV basal diameter of >41 mm or, relatively, as RV diameter greater than or equal to the LV basal diameter. Severe RV systolic dysfunction was determined by visual estimation, tricuspid annular plane systolic excursion less than 10 mm, or interventricular septal position being flat or bending toward the LV base. RV dilatation was considered a requirement for determining RVD. A GDE score was assigned 0 points if no severe RV dilatation was found. A score of 1 point was assigned for severe RV dilatation alone, 2 points were assigned for severe RV dilatation combined with septal deviation or severe RV systolic dysfunction, and 3 points were assigned for severe RV dilatation and both septal deviation and severe RV systolic dysfunction. For this study, RVD was a binary variable and present if GDE score was greater than 0. PE risk stratification was determined using the previously reported PE short-term clinical outcomes risk estimation (PE-SCORE) model, which was developed and validated in the registry database.^12^ The 9-variable points model had scores of 0 to 10 points: Low risk of clinical deterioration = 0, intermediate risk = 1 to 4 points, and high risk = 5 to 10 points. For comparison, we provided the numbers and proportions of our study population considered low risk by two other risk stratification tools: the simplified Pulmonary Embolism Severity Index and the European Society of Cardiology Guidelines.^19,20^

Subsequent rehospitalization was defined as a hospitalization after the index PE hospitalization but within 30 days of PE diagnosis. We considered rehospitalization to be a surrogate for impaired cardiopulmonary functioning or unresolved acute PE-related cardiopulmonary dysfunction.

### Outcome measures

The primary outcome of this report was patient reported QoL. To detect cardiopulmonary fitness and psychosocial impact of recent PE diagnosis, we decided to administer the PEmb-QoL questionnaire assessment 30 ± 3 days after each patient’s enrollment date. Reports on QoL and PE have ranged from 30 days to years after the index PE.^21–23^ We suspect assigning QoL to the end of the expected therapeutic anticoagulation period limits the assessment of PE related QoL to its physiologic treatment. Conducting the QoL assessment 30 days after index PE provides a temporal association of recent PE and QoL and an early opportunity to understand the impact of the PE diagnosis on a patient’s well-being and experiences. Furthermore, a 30-day QoL assessment offers an opportunity for providers and patients to be attuned to issues of compliance and the psychosocial stressors and limitations to well-being, which may be addressed with patients and their support systems. We conducted 30-day QoL assessments prospectively.

A dedicated bilingual research coordinator contacted enrolled patients by telephone and administered the 42-item PEmb-QoL questionnaire. English- and Spanish-speaking patients were enrolled using either the English or Spanish version of the informed consent and the PEmb-QoL participation script, its questions, and its responses. Following a standardized introduction, the sequence and wordings of the questions were systematically performed. There are nine main questions on the original validated PEmb-QoL. We preserved the exact wording of questions from the original PEmb-QoL. We reversed the scores of questions 1, 4, 5, and 9 so that increasing scores were associated with worsened QoL. Questions 2 and 3 were descriptive and therefore not used in scoring reports. Questions 2 and 3 focus on the intensity of lung symptoms and the comparison of past versus current lung symptoms. The absence of current lung symptoms was coded as a missing response. For the domain of work-related problems (question 5), a missing score was used if limitations were not specifically due to lung problems.

The PEmb-QoL was organized into six domains with the accompanying score ranges: frequency of complaints (8 to 40), activities of daily living (12 to 39), work-related problems (4 to 8), social limitations (1 to 5), intensity of complaints for pain (1 to 6) and for breathlessness (1 to 6), and emotional complaints (10 to 60). Total PEmb-QoL score was 37 to 164. Each domain score was also transformed into a 100-point scale. Higher scores signified worse QoL. As reported by Rochat et al.,^24^ we created a summary PEmb-QoL score by averaging the six domain-transformed scores. We used the raw domain and overall scores for univariate analysis. For ease of interpretation, we used rescaling along 100 points for multivariable analysis. This method is often done and has no effect on the conclusions from statistical inference.

### Data analysis

Descriptive statistics, including counts (percentages), means (SDs), and medians (interquartile ranges [IQR]) are reported as appropriate. Univariable analysis of QoL domain and summary scores were compared by predictors, demographic clinical features, comorbid conditions, and echocardiography findings at presentation. We compared the QoL of survivors with abnormalities by GDE who did and did not have reperfusion interventions. Missing data were relatively scarce; we imputed for missing data using imputation by random forest. Imputed values were not used in presenting univariate means and frequencies. We reported missingness for each variable to provide context for how much data was imputed for each variable.

We used raw scores for univariate statistics for each PEmb-QoL domain by our four primary predictors of interest: acute clinical deterioration, RVD (with or without reperfusion intervention within 5 days), PE risk stratification (low-, intermediate-, or high-risk based on PE-SCORE), and subsequent rehospitalization. We used a two-sample *t*-test to compare outcomes by acute clinical deterioration. We used ANOVA to compare outcomes by RVD and by PE-SCORE risk group. The analysis of ordinal variables using statistical methods for continuous variables yields valid inference.^25,26^

We fit independent multivariable linear regression models for each PEmb-QoL domain outcome, as well as a multivariable linear regression model for PEmb-QoL score (the average score for each patient across standardized domain scores on a scale from 0–100). We then fit multivariable regression models for each domain outcome using a more complete set of variables and used least absolute shrinkage and selection operator (LASSO) regression with 10-fold cross-validation for variable selection. We used transformed scores for both unadjusted and LASSO multivariable regressions. The following set of predictors was included in the model: gender, age, presence of any cancer, initial vital signs (including shock index), total Charlson index score, GDE score, length of stay, presence or absence of subsequent rehospitalization, administration of reperfusion intervention, elevated troponin or natriuretic peptide measurements, presence or absence of clinical deterioration within 5 days, PE risk stratification (PE-SCORE), body mass index, presence or absence of coexisting systemic infection, hypovolemia, dysrhythmia, creatinine level > 2.0 mg/dL, severe liver disease, and chronic obstructive pulmonary disease (COPD).

## RESULTS

**FIGURE 1:**
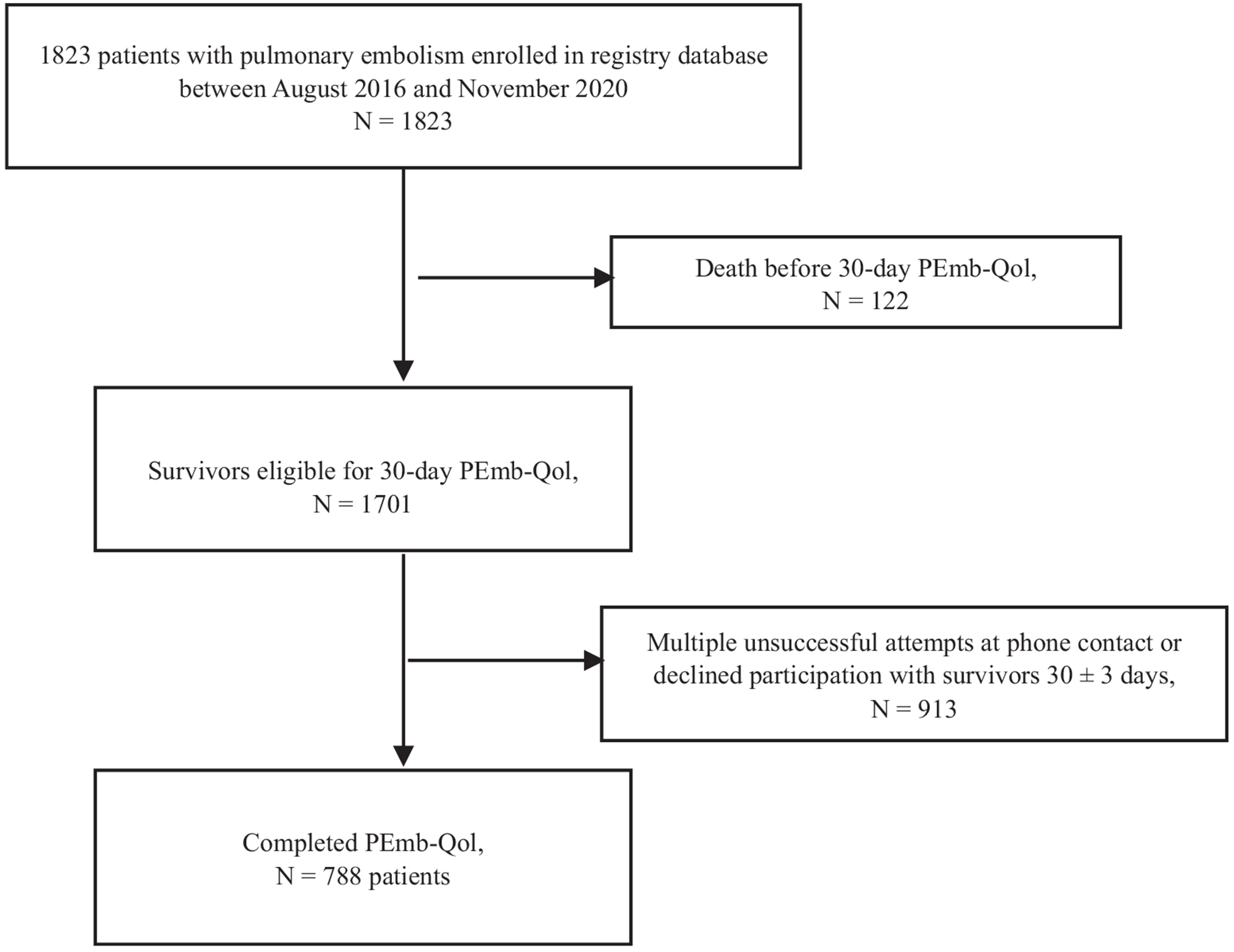
Study flow diagram. PEmb-QoL, Pulmonary Embolism Quality of Life.

We approached 1823 PE patients of any acuity level between August 2016 and November 2020. As shown in Figure 1, complete PEmb-QoL data were available for 788 patients. Of these, 20 patients (2.5%) did not have interpretable GDE images.

Table S1 shows clinical characteristics of the overall study population at index ED evaluation for PE and their PE risk factors. Mean ± SD age was 59 ± 16 years. Patients identified as 48.6% male, 66.9% Caucasian, and 27.9% African American. Table S2 shows characteristics of patients who completed PEmb-QoL (*n* = 788) versus those who did not (*n* = 913). Demographics, vital signs at presentation, and PE risk factors were similar, except the proportion with cancer, which was higher for those who did not complete PEmb-QoL. Table S3 shows results of two nondomain PEmb-QoL questions on when symptoms are most intense and the condition of lungs compared to 1 year ago.

Of the 788 patients, 210 (26.6%) had a previous history of deep vein thrombosis (DVT) or PE (none within the previous 12 months), 49 (6.2%) had recent trauma and injuries, 244 (31.0%) had a recent rehospitalization, 24 (3.0%) had clotting disorder, 157 (19.9%) had cancer, and 39 (4.9%) had acute congestive heart failure (CHF). At the ED presentation, 166 (21.1%) patients were considered low risk by PE-SCORE (PE-SCORE = 0 points). For comparison, 315 (40%) patients were considered low risk by the simplified Pulmonary Embolism Severity Index and 94 (11.9%) were low risk by the European Society of Cardiology Guidelines.

Although 170 of 788 (21.6%) patients were discharged from the ED or hospital within 24 h of PE diagnosis, the mean (±SD) length of stay was 100 (+−118) hours. At hospital admission, 485 (62%) of our PE cohort had DVT investigated, 336 (69.3%) of whom had a concurrent DVT identified. Of the 788 patients, 156 (19.8%) experienced one or more clinical deterioration events within 5 days of PE diagnosis, including cardiac arrest in seven (0.9%), respiratory failure in 45 (5.8%), hypotension requiring pressors in 26 (3.3%), new dysrhythmia in 43 (5.5%), and reperfusion intervention in 38 (4.8%).

Of the 788 patients with complete PEmb-QoL data, 785 (99.1%) received anticoagulation, 762 (97.1%) of whom were compliant 1 month later. Of 768 patients with complete PEmb-QoL data and interpretable GDE images, 236 (30.7%) had abnormal RV features. Of the 236 patients, 38 (16.1%) patients received escalated treatment with thrombolysis or embolectomy. Of 529 patients without abnormal RV by GDE at presentation, seven (1.9%) received escalated PE intervention. Of the 788 patients, 87 (11%) had subsequent rehospitalization within the 30-day period.

### Univariable analysis results

Table 1 shows there were only a few statistically significant differences between any of the six PEmb-QoL domain scores of patients with and without clinical deterioration within 5 days. The exception was social limitations (question 6), with mean (±SD) scores of 2.07 (±1.27) and 2.36 (±1.47), respectively (*p* = 0.027). Mean (±SD) overall PEmb-QoL scores for those with and without clinical deterioration were 33.4 (±22.4) and 29.8 (±22.0), respectively (*p* = 0.083).

For RVD by GDE with or without reperfusion (reference group: no RVD by GDE), Table 2 shows the only difference in mean do-main scores between groups were for questions 7 and 8 (intensity of complaints about pain and breathlessness). For intensity of complaints, mean (±SD) scores for patients without RVD 4.32 (±2.69) were higher (worse) than for those with RVD (3.82 [±1.81] and 3.83 [±2.11] with and without reperfusion intervention, respectively).

Table 3 shows PEmb-QoL domain score stratified by initial PE-SCORE risk classification. None of the domains had significant differences between low-, intermediate-, and high-risk PE-SCORE groups. Table 4 shows all PEmb-QoL domain scores and total scores were significantly worse for patients with subsequent rehospitalization versus those without.

### Multivariable analysis results

Tables S4–S9 show multivariable linear regression effect size estimates for each PEmb-QoL domain score (transformed on a scale from 0–100). Subsequent rehospitalization and length of stay were significantly associated with higher QoL scores.

Table 5 shows effect size estimates of key predictors of interest for overall PEmb-QoL scores. Statistical significance was present if the 95% CI did not include 0 points. Subsequent rehospitalization (12.58 points [95% CI 7.86–17.30]) and longer length of stay (0.06 [95% CI 0.03–0.09]) were associated with higher PEmb-QoL scores. In contrast, the estimated effect sizes for acute clinical deterioration and PE-SCORE were 1.02 (−3.47 to 5.52) points and −0.02 (−1.47 to 1.42) points, respectively. RVD by GDE with or without reperfusion compared to no RVD by GDE showed nonsignificant effect size estimates of −5.67 and −3.55 points lower, respectively.

Table 5 also shows the retained variables after the LASSO regression for overall PEmb-QoL scores and their effect sizes. COPD, cancer, increased length of stay, and subsequent rehospitalization within 30 days had significant effect sizes. Patients with COPD had PEmb-QoL scores 8.17 points higher (95% CI 3.91–12.43) than patients without COPD. Patients with any cancer had PEmb-QoL scores 5.02 points *lower than* patients without any cancer. Subsequent rehospitalization had effect size 11.29 (95% CI 6.68–15.89).

## DISCUSSION

We prospectively investigated whether predictors of clinical deterioration and the occurrence of clinical deterioration itself were associated with patient reported QoL 1 month after a PE. In our database of 788 PE survivors, few demographic, clinical, and physiologic variables at presentation of the acute index PE event were associated with scores on PE QoL domains conducted 30 ± 3 days later. Acute clinical deterioration, RVD status, and PE risk stratification were not significant for PEmb-QoL scores 1 month later. We found COPD, length of stay, and subsequent rehospitalization were associated with worse QoL reports. Rehospitalizations within 30 days may signal a negative impact of the index PE on QoL.

Clinical deterioration, initial RVD status, and PE risk stratification at presentation are all important, but they may not address the patient’s level of functioning and adjustments to life after index PE. QoL is an important endpoint that addresses a patient’s impression of the impact of the disease and the efficacy of treatment interventions on their well-being. Although there are many studies on the effects of DVT on QoL, there are few studies on patient-reported QoL within 1 month of PE.^6^ There are reports that determine the effect of escalated treatments beyond anticoagulation for patients with PE and RVD compared to standard anticoagulation. These studies show persistence of RVD on echocardiography and reduced performance on 6-minute American Heart Association walk test when tested 6 months later; however, patient QoL was not an outcome measure.^9,27^ Our results suggest decreased QoL after PE may occur despite initial RVD status. QoL scores were not significantly different between PE patients with RVD who had escalated PE interventions versus those who did not. Early rehospitalization may be a surrogate sign associated with a negative psychosocial impact of the PE experience.

Although the causes of both PE and DVT are similar, the long-term complications of PE and DVT are different. PE may occur with or without an identifiable or concurrent DVT. Post-DVT syndromes are associated with more work-related disabilities than post-PE syndromes. In a study by Braekkan et al.,^28^ subjects with DVT had a 52% higher incidence of work-related disability, but there was no increase in work-related disability after PE. Although we are using the PEmb-QoL instrument, the impact on QoL may be affected by multiple variables including postthrombotic syndrome, VTE recurrence, and chronic pulmonary hypertension. Chronic pulmonary hypertension, however, is less common after PE than postthrombotic syndrome is after DVT.

The most common outcomes studied for PE risk stratification are death, recurrence of PE, and bleeding. More recently, short-term clinical deterioration after diagnosis has received attention now that novel oral anticoagulants have become available.^15^ However, there are only a few reports on the variables associated with and predictive of PE-specific QoL.^21^ There are several plausible factors that could influence post-VTE disability. These factors include, but are not limited to, the presence of comorbid conditions that are discovered with or worsened by the index PE, recurrence of either DVT or PE, recent traumatic injuries provoking the PE and limiting and challenging recovery to full premorbid functional status, and compliance and efficacy of anticoagulation therapy.

Comorbidities may influence QoL after PE. In a recent study by van Es et al.,^29^ a cohort of 109 outpatients treated for PE reported lower QoL compared to the general population. COPD, CHF, and cancer were correlated with worse QoL scores. Similarly, Klok et al.^22^ used an SF-36 QoL tool and reported lower QoL was related to age, obesity, cancer, and cardiopulmonary comorbid conditions in their study.

We found COPD, length of stay, and subsequent rehospitalization were associated with worse QoL 1 month later. Although statistically significant, the effect size estimate on PEmb-QoL for length of stay (0.06 points) was minimal. In contrast, the effect size estimates for COPD (8.17 points) and subsequent rehospitalization (11.29 points) were substantial. COPD and subsequent rehospitalizations may be related or interact. Anxiety about cardiopulmonary symptoms of COPD after PE may lead to repeat ED visits and rehospitalizations. Preexisting COPD and recent PE may compromise cardiopulmonary fitness and negatively impact QoL.

Work-related dysfunction can lead to economic burdens to individual patients (lost workdays and lost wages) but also substantial costs to the patient’s own circle of support and the patient’s dependents. In our study, increased length of hospital stay and subsequent rehospitalizations within 30 days were associated with worse QoL scores.

The clinical relevance of the study is that it challenges the current focus on cardiopulmonary predictors (vital signs and cardiac function) and outcomes (death, clinical deterioration, recurrence of PE, and major bleeding). Predictors employed in risk stratification for disposition decisions and early phase/outpatient clinical management currently focus on cardiopulmonary functioning and safety of anticoagulation. Our study results challenge the current paradigm.

Compared to the earlier year, our patients reported reduced QoL 1 month after the index PE. We analyzed the association between clinical testing and clinical outcomes, which are considered pertinent to the early phases of PE care, to subsequent patient-reported QoL. We found a strong association between comorbidities and reduced QoL. We also found an insignificant relationship between markers of PE severity, such as abnormal RV and subsequent clinical deterioration, with reduced QoL. Therefore, it is plausible that a PE diagnosis may be associated with reduced QoL for any patient regardless of the degree of PE severity.

Our report suggests psychosocial stressors should be considered as both predictors and outcomes by health care providers. With this insight, health care providers may offer different conversations and resources to patients during hospitalization and outpatient follow-up. Further stringent inquiry into determinants of QoL may frame and advise the counseling and monitoring of patients with PE.

## LIMITATIONS

This study had several limitations. First, the proportion of survivors not participating in the QoL assessment was high (53.4%). As previously reported, the research registry and database focused on ED patients diagnosed with acute symptomatic PE with concurrent RV computed tomography and echocardiography imaging, electrocardiography, and measurement of cardiac biomarkers.^12,30,31^ The primary outcomes of the research registry were clinical deterioration events within 5 days and 30 days. We did not exclude patients from the registry database if they did not participate in the 30-day QoL assessment. A report on 30-day QoL assessments was planned. For this current report, the focus was on those participating in PEmb-QoL. Instead of reporting on a convenience sample, Table S2 disclosed a side-by-side comparison of the early characteristics of patients who did and did not participate in the QoL assessment. We contacted patients by telephone days before PEmb-QoL administration and within the 6-day window. Our capture of eligible patients is similar to other studies, which report 50%–75% participation, even in low-risk PE patients.^3,32^ In retrospect, we should have included strategies for successful follow-up and PEmb-QoL completion. Recent QoL studies have provided prepaid mobile phones to patients without telephones for research purposes, conducted the PEmb-QoL via virtual video conference, or provided an electronic questionnaire via email.^3,33^

Second, a single PEmb-QoL was performed 30 days after the index PE event. We were not aware of the patients’ baseline cardiopulmonary symptoms, fitness or function, nor their mental health or social/work limitations. This pre-PE information was not available nor feasible in this study design. Therefore, we were not able to identify the reason cancer was associated with better QoL scores 1 month post-PE than no cancer. Although cancer is a known risk factor for VTE, cancer itself may not influence the severity of PE or the susceptibility of the PE to standard treatments. It is also plausible that patient reported QoL domains for a patient with cancer may not be impacted by the new diagnosis of PE. Regardless, the association warrants further investigation.

Third, our choice to conduct one QoL assessment 1 month after PE presents limitations. One month may not be a long enough time; however, we believe there is value to early identification of reduced QoL and that our study will fill a gap in the literature. Most PE or DVT-specific QoL assessments in the literature have been conducted between 10 and 24 months after the index event.^6,10,11,23,32–34^ An advantage of a shorter interval is better patient recall. A disadvantage of assessing QoL 1 month after a PE is the patient most likely has not completed the prescribed anticoagulation treatment, which is usually at least 6 weeks long, and thus is still recovering from their PE. However, it is plausible that early assessment of QoL may reveal slower than expected recovery and signal interventions to reduce the persistence and severity of disability.^7^

We recognize serial QoL assessments might reveal different predictors of QoL beyond 1 month. In one study, PEmb-QoL scores for low-risk PE patients improved from 3 weeks post-PE to 3 months post-PE.^3^ Another study that included serial PEmb-QoL assessments (baseline plus 1, 3, and 6 months and 1 year after the index PE event) showed improvements in QoL over the year.^15^

Fourth, it is plausible that PEmb-QoL in its current form may not represent recently treated PE patients. The PEmb-QoL was developed after semistructured interviews of 10 patients who suffered PE between 7 months and 7 years before the interview. In the original report, details on the treatment course of these 10 patients were not provided.^10^

Fifth, PEmb-QoL uses structured questions and responses that may limit a patient’s description and expressions of their experiences and feelings after PE. PEmb-QoL places an emphasis on lung symptoms in several of the structured questions. Patients may experience concerns other than lung symptoms (e.g., palpitations, anxiety, dizziness). Fears of recurrence may limit activities due to concerns of injuries that lead to immobilization or bleeding complications. Likewise, a questionnaire has limits that interviews may overcome. The interpretation of the patient’s experience is limited by the choices made and does not allow for the interviewer’s interpretation of collateral comments, which point to specific responses or correct selections made by the patient. In contrast, semistructured questions posed in interviews allow for real-world conversational expressions. Follow-up questions and responses allow patients to expound their responses, potentially revealing nuances and richer information that structured responses may miss.^33^ The structured PEmb-QoL questionnaire does have some advantages, though, over semistructured interviews, including objectivity, better time to completion, and the avoidance of biased ratings by interviewers. These advantages are helpful in clinical trials and large observational studies.^5,6^

Sixth, we assessed the association of early echocardiography findings of RV dilatation or systolic dysfunction with QoL. We did not repeat echocardiography assessments of survivors. It is plausible that subsequent abnormal findings may have persisted, worsened, or improved as the condition evolved. In addition, the magnitude and direction of any changes from baseline echocardiography findings may be associated with QoL experiences reported by the patient.

Seventh, the strong associations of length of stay and subsequent rehospitalization with PEmb-QoL scores may not be causal. It is plausible that deconditioning may occur with longer hospitalizations or comorbid conditions like COPD that lead to increased susceptibility to PE recurrence or prolonged recovery from the index PE. It is plausible that length of stay and subsequent rehospitalizations are surrogate predictors of worse QoL.

Finally, our study did not include data collection on the presence or absence of conversations between health care providers and patients (during index PE hospitalization or postdischarge follow-up) or patients’ understanding about the illness, its treatment, and guidance with monitoring of fitness. These conversations may have a psychosocial impact on QoL and subsequent rehospitalizations.

## CONCLUSIONS

Thirty days after the index pulmonary embolism, our patients reported worse QoL than the previous year. Neither acute clinical deterioration (within 5 days of index pulmonary embolism) nor echocardiography assessment during ED presentation showing right ventricular dysfunction were significant predictors of QoL 1 month post–pulmonary embolism. Chronic obstructive pulmonary disease, subsequent rehospitalization, and increased length of stay were associated with worse patient-reported QoL by survivors 1 month after index pulmonary embolism diagnosis. It is possible the diagnosis of pulmonary embolism may be associated with reduced quality of life for any patient regardless of the degree of pulmonary embolism severity.

## Supplementary Material

Table S1

Supplementary Index

Table S2

Table S3

Table S4

Table S5

Table S6

Table S7

Table S8

Table S9

## Figures and Tables

**TABLE 1:** Univariate statistics for each domain score by acute clinical deterioration.

	Acute clinical deterioration			
PEmb-QoL domains	No (*n* = 632)	Yes (*n* = 156)	Overall (*n* = 788)	*p*-value
Frequency of complaints				
Mean (±SD)	12.8 (±6.10)	12.8 (±6.73)	12.8 (±6.22)	0.964
Median [min, max]	11.0 [8.00, 40.0]	10.5 [8.00, 40.0]	11.0 [8.00, 40.0]	
Missing, *n* (%)	6.00 (0.9%)	0 (0%)	6.00 (0.8%)	

Activities of daily living				
Mean (±SD)	21.8 (±7.94)	23.1 (±8.13)	22.0 (±7.99)	0.073
Median [min, max]	20.0 [12.0, 39.0]	24.0 [12.0, 39.0]	21.0 [12.0, 39.0]	
Missing, *n* (%)	31.0 (4.9%)	14.0 (9.0%)	45.0 (5.7%)	

Work-related problems				
Mean (±SD)	6.19 (±1.81)	6.41 (±1.81)	6.23 (±1.81)	0.173
Median [min, max]	7.00 [4.00, 8.00]	8.00 [4.00, 8.00]	7.00 [4.00, 8.00]	
Missing, *n* (%)	6.00 (0.9%)	0 (0%)	6.00 (0.8%)	

Social limitations				
Mean (±SD)	2.07 (±1.27)	2.36 (±1.47)	2.12 (±1.32)	0.027
Median [min, max]	2.00 [1.00, 5.00]	2.00 [1.00, 5.00]	2.00 [1.00, 5.00]	
Missing, *n* (%)	8.00 (1.3%)	4.00 (2.6%)	12.0 (1.5%)	

Intensity of complaints				
Mean (±SD), *n* (%)	4.17 (±2.54)	4.20 (±2.45)	4.18 (±2.52)	0.9
Median [min, max]	4.00 [2.00, 20.0]	4.00 [2.00, 11.0]	4.00 [2.00, 20.0]	
Missing, *n* (%)	6.00 (0.9%)	0 (0%)	6.00 (0.8%)	

Emotional complaints				
Mean (±SD)	19.6 (±9.97)	20.3 (±10.3)	19.7 (±10.0)	0.475
Median [min, max]	16.0 [10.0, 58.0]	17.0 [10.0, 58.0]	16.0 [10.0, 58.0]	
Missing, *n* (%)	14.0 (2.2%)	3.00 (1.9%)	17.0 (2.2%)	

PEmb-QoL score average				
Mean (±SD)	29.8 (±22.0)	33.4 (±22.4)	30.5 (±22.2)	0.083
Median [min, max]	27.6 [0, 101]	32.9 [0, 84.5]	28.8 [0, 101]	
Missing, *n* (%)	53.0 (8.4%)	16.0 (10.3%)	69.0 (8.8%)	

Length of stay (h), mean (±SD)	81.82 (±96.18)	167.61 (±155.09)	98.82 (±115.45)	<0.001

Abbreviation: PEmb-QoL, Pulmonary Embolism Quality-of-Life questionnaire.

**TABLE 2:** Univariate statistics for each domain score by RVD with or without intervention.

GDE findings and treatment	No RVD (*N* = 532)	RVD with reperfusion (*N* = 38)	RVD without reperfusion (*N* = 198)	Overall (*N* = 788)	*p*-value
Frequency of complaints					
Mean (±SD)	13.1 (±6.67)	11.6 (±3.75)	12.2 (±5.36)	12.8 (±6.22)	0.076
Missing, *n* (%)	6.00 (1.1%)	0 (0%)	0 (0%)	6.00 (0.8%)	

Activities of daily living					
Mean (±SD)	22.1 (±8.09)	21.5 (±8.10)	22.0 (±7.64)	22.0 (±7.99)	0.929
Missing, *n* (%)	26.0 (4.9%)	1.00 (2.6%)	17.0 (8.6%)	45.0 (5.7%)	

Work-related problems					
Mean (±SD)	6.27 (±1.81)	6.24 (±1.95)	6.18 (±1.79)	6.23 (±1.81)	0.847
Missing, *n* (%)	4.00 (0.8%)	0 (0%)	2.00 (1.0%)	6.00 (0.8%)	

Social limitations					
Mean (±SD)	2.13 (±1.34)	2.35 (±1.44)	2.08 (±1.26)	2.12 (±1.32)	0.51
Missing, *n* (%)	7.00 (1.3%)	1.00 (2.6%)	3.00 (1.5%)	12.0 (1.5%)	

Intensity of complaints					
Mean (±SD)	4.32 (±2.69)	3.82 (±1.81)	3.83 (±2.11)	4.18 (±2.52)	0.043

Emotional complaints					
Mean (±SD)	19.9 (±10.2)	18.8 (±10.3)	19.5 (±9.72)	19.7 (±10.0)	0.765
Missing, *n* (%)	11.0 (2.1%)	0 (0%)	6.00 (3.0%)	17.0 (2.2%)	

PEmb-QoL score average					
Mean (±SD)	31.2 (±22.8)	29.6 (±20.7)	28.9 (±20.6)	30.5 (±22.2)	0.48
Missing, *n* (%)	45.0 (8.5%)	2.00 (5.3%)	21.0 (10.6%)	69.0 (8.8%)	

Abbreviations: GDE, goal-directed echocardiography; PEmb-QoL, pulmonary embolism quality-of-life questionnaire; RVD, right ventricular dysfunction.

**TABLE 3:** Univariate statistics for each domain score by PE-SCORE risk class.

	PE-SCORE risk classifications				
PEmb-QoL domains	Low (0) (*n* = 166)	Intermediate (1–4) (*n* = 529)	High (5–10) (*n* = 48)	Overall (*n* = 788)	*p*-value
Frequency of complaints					
Mean (±SD)	13.4 (±5.56)	12.7 (±6.49)	11.6 (±5.99)	12.8 (±6.22)	0.174
Median [min, max]	12.0 [8.00, 29.0]	11.0 [8.00, 40.0]	9.50 [8.00, 38.0]	11.0 [8.00, 40.0]	
Missing, *n* (%)	2.00 (1.2%)	4.00 (0.8%)	0 (0%)	6.00 (0.8%)	

Activities of daily living					
Mean (±SD)	21.1 (±7.77)	22.4 (±8.03)	22.0 (±7.87)	22.0 (±7.99)	0.212
Median [min, max]	19.0 [12.0, 39.0]	21.0 [12.0, 39.0]	20.5 [12.0, 36.0]	21.0 [12.0, 39.0]	
Missing, *n* (%)	4.00 (2.4%)	29.0 (5.5%)	8.00 (16.7%)	45.0 (5.7%)	

Work-related problems					
Mean (±SD)	6.05 (±1.82)	6.35 (±1.80)	5.96 (±1.91)	6.23 (±1.81)	0.093
Median [min, max]	6.00 [4.00, 8.00]	7.00 [4.00, 8.00]	5.50 [4.00, 8.00]	7.00 [4.00, 8.00]	
Missing, *n* (%)	0 (0%)	6.00 (1.1%)	0 (0%)	6.00 (0.8%)	

Social limitations					
Mean (±SD)	1.96 (±1.19)	2.20 (±1.36)	2.04 (±1.36)	2.12 (±1.32)	0.115
Median [min, max]	1.00 [1.00, 5.00]	2.00 [1.00, 5.00]	1.00 [1.00, 5.00]	2.00 [1.00, 5.00]	
Missing, *n* (%)	1.00 (0.6%)	6.00 (1.1%)	3.00 (6.3%)	12.0 (1.5%)	

Intensity of complaints					
Mean (±SD)	4.27 (±2.57)	4.16 (±2.52)	3.79 (±2.41)	4.18 (±2.52)	0.509
Median [min, max]	4.00 [2.00, 20.0]	4.00 [2.00, 15.0]	2.50 [2.00, 11.0]	4.00 [2.00, 20.0]	
Missing, *n* (%)	1.00 (0.6%)	5.00 (0.9%)	0 (0%)	6.00 (0.8%)	

Emotional complaints					
Mean (±SD)	20.1 (±9.87)	19.8 (±10.1)	18.9 (±10.8)	19.7 (±10.0)	0.778
Median [min, max]	17.0 [10.0, 57.0]	16.0 [10.0, 58.0]	14.5 [10.0, 55.0]	16.0 [10.0, 58.0]	
Missing, *n* (%)	3.00 (1.8%)	12.0 (2.3%)	2.00 (4.2%)	17.0 (2.2%)	

PEmb-QoL score average					
Mean (±SD)	28.9 (±21.5)	31.3 (±22.2)	29.1 (±23.7)	30.5 (±22.2)	0.446
Median [min, max]	27.1 [0, 101]	29.7 [0, 96.5]	26.3 [0, 81.3]	28.8 [0, 101]	
Missing, *n* (%)	11.0 (6.6%)	44.0 (8.3%)	9.00 (18.8%)	69.0 (8.8%)	

Abbreviations: PEmb-QoL, Pulmonary Embolism Quality-of-Life questionnaire; PE-SCORE, pulmonary embolism short-term clinical outcomes risk estimation.

**TABLE 4:** Univariate statistics for each domain score by subsequent rehospitalization.

PEmb-QoL domains	No subsequent rehospitalization (*n* = 701)	Subsequent rehospitalization (*n* = 87)	Overall (*n* = 788)	*p*-value
Frequency of complaints				
Mean (±SD)	12.5 (±5.67)	15.7 (±9.14)	12.8 (±6.22)	0.002
Median [min, max]	11.0 [8.00, 40.0]	12.0 [8.00, 40.0]	11.0 [8.00, 40.0]	
Missing, *n* (%)	5 (0.7%)	1 (1.1%)	6 (0.8%)	

Activities of daily living				
Mean (±SD)	21.6 (±7.89)	25.7 (±7.90)	22.0 (±7.99)	<0.001
Median [min, max]	20.0 [12.0, 39.0]	26.5 [12.0, 39.0]	21.0 [12.0, 39.0]	
Missing, *n* (%)	40 (5.7%)	5 (5.7%)	45 (5.7%)	

Work-related problems				
Mean (±SD)	6.15 (±1.82)	6.94 (±1.61)	6.23 (±1.81)	<0.001
Median [min, max]	7.00 [4.00, 8.00]	8.00 [4.00, 8.00]	7.00 [4.00, 8.00]	
Missing, *n* (%)	4 (0.6%)	2 (2.3%)	6 (0.8%)	

Social limitations				
Mean (±SD)	2.06 (±1.27)	2.64 (±1.57)	2.12 (±1.32)	0.002
Median [min, max]	2 [1.00, 5.00]	3 [1.00, 5.00]	2.00 [1.00, 5.00]	
Missing, *n* (%)	10 (1.4%)	2 (2.3%)	12 (1.5%)	

Intensity of complaints				
Mean (±SD)	4.04 (±2.32)	5.34 (±3.60)	4.18 (±2.52)	0.002
Median [min, max]	4.00 [2.00, 12.0]	5.00 [2.00, 20.0]	4.00 [2.00, 20.0]	
Missing, *n* (%)	4 (0.6%)	2 (2.3%)	6 (0.8%)	

Emotional complaints				
Mean (±SD)	19.3 (±9.77)	23.3 (±11.3)	19.7 (±10.0)	0.002
Median [min, max]	16.0 [10.0, 58.0]	20.0 [10.0, 58.0]	16.0 [10.0, 58.0]	
Missing, *n* (%)	15 (2.1%)	2 (2.3%)	17 (2.2%)	

PEmb-QoL score average				
Mean (±SD)	29.0 (±21.3)	42.8 (±25.1)	30.5 (±22.2)	<0.001
Median [min, max]	27.1 [0, 96.5]	41.6 [0.333, 101]	28.8 [0, 101]	
Missing, *n* (%)	63 (9.0%)	6 (6.9%)	69 (8.8%)	

Abbreviation: PEmb-QoL, Pulmonary Embolism Quality-of-Life questionnaire.

**TABLE 5:** Multivariable linear regression and variable selection models with estimates of effect sizes for overall PEmb-QoL.

Predictors	Estimates	CI	*p* value
Multivariable linear regression			
(Intercept)	24.02	21.29 to 26.75	<0.001
PE-SCORE points	−0.02	−1.47 to 1.42	0.976
Acute clinical deterioration	1.02	−3.47 to 5.52	0.655
RVD by GDE with reperfusion intervention	−5.67	−14.14 to 2.79	0.189
RVD by GDE without reperfusion intervention	−3.55	−8.18 to 1.07	0.132
Subsequent rehospitalization	12.58	7.86 to 17.30	<0.001
Length of stay	0.06	0.03 to 0.09	<0.001
R2/R2 adjusted	0.067/0.059		

Multivariable regression with variable selection (LASSO) for overall score			
(Intercept)	42.04	25.43 to 58.65	<0.001
Gender	6.18	3.31 to 9.05	<0.001
Age	−0.21	−0.31 to −0.12	<0.001
Cancer	−5.02	−9.87 to −0.18	0.042
Initial systolic pressure (mmHg)	−0.08	−0.16 to 0.00	0.059
Initial shock index	−12.38	−20.83 to −3.93	0.004
Initial respiratory rate	0.34	−0.05 to 0.72	0.084
Total Charlson index	1.44	0.49 to 2.38	0.003
Length of stay	0.06	0.03 to 0.08	<0.001
Subsequent rehospitalization	11.29	6.68 to 15.89	<0.001
COPD	8.17	3.91 to 12.43	<0.001
R2/R2 adjusted	0.140/0.129		

Abbreviations: COPD, chronic obstructive pulmonary disease; GDE, goal-directed echocardiography; LASSO, least absolute shrinkage and selection operator; PEmb-QoL, Pulmonary Embolism Quality-of-Life questionnaire; PE-SCORE, pulmonary embolism short-term clinical outcomes risk estimation; RVD, right ventricular dysfunction.
