# Supplementary material for: Quality of life 1 month after acute pulmonary embolism in emergency department patients
Source: Acad Emerg Med. Author manuscript; Available in PMC 2025 Apr 5. (PMC11971718; doi:10.1111/acem.14692)
Supplement: Table S1 [file NIHMS2065999-supplement-Table_S1.pdf]

Table S1: Univariate statistics for all variables*

	Score range	Overall (N = 788)
**Frequency of Complaints**	8 to 40	
Mean (SD)		12.8 (6.22)
Missing		6 (0.8%)
**Activities of Daily Living**	12 to 39	
Mean (SD)		22 (7.99)
Missing		45 (5.7%)
**Work-related Problems**	4 to 8	
Mean (SD)		6.23 (1.81)
Missing		6 (0.8%)
**Social Limitations**	1 to 5	
Mean (SD)		2.12 (1.32)
Missing		12 (1.5%)
**Intensity of Complaint: Pain**	1 to 6	
Mean (SD)		1.81 (1.37)
Missing		2.00 (0.3%)
**Intensity of Complaint: Breathlessness**	1 to 6	
Mean (SD)		2.36 (1.60)
Missing		3.00 (0.4%)
**Intensity of Complaints (combined pain and breathlessness)**	2 to 12	
Mean (SD)		4.18 (2.52)
Median [Min, Max]		4.00 [2.00, 20.0]
Missing		6.00 (0.8%)

**Emotional Complaints**	10 to 60	
Mean (SD)		19.7 (10.0)
Missing		17.0 (2.2%)
**PEmb-QoL score average**	37 to 164	
Mean (SD)		30.5 (22.2)
Missing		69 (8.8%)

**GDE showing RVD?**	
No	532 (67.5%)
Yes	236 (29.9%)
Missing	20 (2.5%)
**Reperfusion intervention within 5 days**	
Yes	44.0 (5.6%)
**Anticoagulation compliance at 30 days**	
No	26 (3.3%)
Yes	759 (96.3%)
Missing	3 (0.4%)
**Pre-existing or new disability that changes the outcome of the questionnaire**	
No	671 (85.2%)
Yes	93 (11.8%)
Missing	24 (3.0%)
**Prior diagnosis of PE or DVT**	
Yes	210 (26.6%)
**Recent trauma**	
Yes	49.0 (6.2%)
Missing	1 (0.1%)
**Recent hospitalization?**	
Yes	244 (31.0%)

**Clotting disorders (protein c, s, factor V...)**	
Yes	24 (3.0%)
Missing	1 (0.1%)
**Sex**	
Female	405 (51.4%)
Male	383 (48.6%)
**Race**	
African American	220 (27.9%)
Caucasian	527 (66.9%)
Asian	9 (1.1%)
Native American	5 (0.8%)
Other	21 (2.7%)
Unknown	5 (0.6%)
**Ethnicity**	
Hispanic	56 (7.1%)
Not Hispanic	707 (89.7%)
Unanswered	25 (3.2%)
**Age, years**	
Mean (SD)	58.9 (15.9)
**Any cancer?**	
Yes	157 (19.9%)
**Low risk sPESI?**	
Yes	315 (40.0%)
**PE-SCORE points assigned**	
0	166 (21.1%)
1 to 4 points	529 (67.1.%)
5 to 10 points	48 (6.1%)
Missing	45 (5.7%)

**Low risk ESC?**	
No	694 (88.1%)
Yes	94 (11.9%)
**Natriuretic peptide elevation**	
Yes	309 (39.2%)
Missing	39 (4.9%)
**Troponin elevation**	
Yes	196 (24.9%)
Missing	12 (1.5%)
**Suspected/confirmed severe LV dysfunction?**	
Yes	32 (4.1%)
Missing	2 (0.3%)
**DVT ultrasound findings if assessed**	
No	137 (17.4%)
Yes	334 (42.4%)
Not evaluated	317 (40.2%)
**Acute clinical deterioration within 5 days?**	
No	632 (80.2%)
Yes	156 (19.8%)
**Cardiac arrest within 5 days**	
No	781 (99.1%)
Yes	7 (0.9%)
**Respiratory failure within 5 days**	
No	742 (94.2%)
Yes	46 (5.8%)
**Dysrhythmia within 5 days**	
No	745 (94.5%)
Yes	43 (5.5%)

**Hypotension_pressors within 5 days**	
No	762 (96.7%)
Yes	26 (3.3%)
**Hypotension_fluid within 5 days**	
No	732 (92.9%)
Yes	56 (7.1%)
**Recurrence of VTE within 30 days**	
No	780 (99.0%)
Yes	8 (1.0%)
**Major bleeding within 30 days**	
No	767 (97.3%)
Yes	21 (2.7%)
**Systolic blood pressure, mmHg**	
Mean (SD)	134 (24.4)
**Heart rate, beats per minute**	
Mean (SD)	96.5 (21.4)
**Shock index**	
Mean (SD)	0.75 (0.240)
**Respiratory rate, breaths per minute**	
Mean (SD)	19.7 (3.94)
**Spontaneous breathing?**	
No	29 (3.7%)
Yes	759 (96.3%)
**O2 Saturation**	
Mean (SD)	95.6 (4.11)
**Respiratory rate >30 bpm**	
Yes	26 (3.3%)
**Temperature**	

Mean (SD)	98.2 (0.886)
**Transient hypotension prior to enrollment**	
Yes	53 (6.7%)
**Preceding episodes of syncope**	
Yes	66 (8.4%)
**Preceding episodes bradycardia? (< 40 bpm)**	
No	777 (98.6%)
Yes	11.0 (1.4%)
**Family history of VTE**	
0	732 (92.9%)
Yes	56 (7.1%)
**Hormone replacement therapy**	
Yes	25 (3.2%)
**Indwelling catheter**	
Yes	44 (5.6%)
**BMI**	
Mean (SD)	32.6 (9.40)
**BMI > 30**	
Yes	422 (53.6%)
**Suspected/confirmed systemic infection**	
Yes	41.0 (5.2%)
**Severe renal impairment? (creatinine > 2.0)**	
Yes	25 (3.2%)
**Severe liver impairment?**	
Yes	8 (1.0%)
Missing	4 (0.5%)
**Chronic pulmonary disease**	
Yes	119 (15.1%)

**AIDS (not just HIV positive)**	
0	783 (99.4%)
1	3 (0.4%)
Missing	2 (0.3%)
**Total Charlson index**	
0	361 (45.8%)
1	143 (18.1%)
2	112 (14.2%)
3	57.0 (7.2%)
4	23.0 (2.9%)
5	21.0 (2.7%)
6	36.0 (4.6%)
7	18.0 (2.3%)
8	6.00 (0.8%)
9	5.00 (0.6%)
10	6.00 (0.8%)
**Is CT RV/LV ratio 1.0 or more**	
No	506 (64.2%)
Yes	265 (33.6%)
Missing	17 (2.2%)
**GDE Score 0–3**	
0	532 (67.5%)
1	57 (7.2%)
2	97 (12.3%)
3	82 (10.4%)
Missing	20 (2.5%)
**Anticoagulant initiated in ED?**	
No	47 (6.0%)

Yes	739 (93.8%)
Missing	2 (0.3%)
**Active bleeding or high risk of bleeding including high risk post-operative state?**	
0	711 (90.2%)
1	69 (8.8%)
Missing	8 (1.0%)
**Oxygen supply to maintain oxygen saturation > 90% for > 24 hours?**	
0	486 (61.7%)
1	299 (37.9%)
Missing	3.00 (0.4%)
**Pulmonary embolism diagnosed during anticoagulation treatment or with INR > 1.7?**	
0	728 (92.4%)
1	55 (7.0%)
Missing	5 (0.6%)
**Intractable pain?**	
0	763 (96.8%)
1	22.0 (2.8%)
Missing	3.00 (0.4%)
**Medical or social reason for treatment in the hospital > 24 hours?**	
No	391 (49.6%)
Yes	392 (49.7%)
Missing	5 (0.6%)
**Length of stay, hours**	
Mean (SD)	100 (118)
**GDE showing RVD with or without reperfusion**	
No	532 (67.5%)

Yes with reperfusion intervention	38 (4.8%)
Yes without reperfusion intervention	198 (25.1%)
Missing	20 (2.5%)

* Abbreviations: PEmb-QoL = pulmonary embolism quality of life questionnaire, GDE = goal directed echocardiography, RVD = right ventricular dysfunction, PE = pulmonary embolism, DVT = deep venous thromboembolism, sPESI = simplified pulmonary embolism severity index, PE-SCORE = pulmonary embolism short-term clinical outcomes risk estimation, ESC = European Society of Cardiology, LV = left ventricle, VTE = venous thromboembolism, BMI = body mass index, AIDS = acquired immunodeficiency syndrome, HIV = human immunodeficiency virus, CT = computed tomography, ED = emergency department.
