# Supplementary material for: Quality of life 1 month after acute pulmonary embolism in emergency department patients
Source: Acad Emerg Med. Author manuscript; Available in PMC 2025 Apr 5. (PMC11971718; doi:10.1111/acem.14692)
Supplement: Supplementary Index [file NIHMS2065999-supplement-Supplementary_Index.pdf]

Supplementary Index

- **Table S1:** Univariate statistics for all variables (PDF, 9 pages, 133 KB)
- **Table S2:** Characteristics of those completing PEmb-QoL versus those who did not (PDF, 7 pages, 66 KB)
- **Table S3:** Results of two non-domain PEmb-QoL questions (PDF, 2 pages, 17 KB)
- **Table S4:** Multivariable analyses of predictors of Frequency of complaints domain score (PDF, 1 page, 66 KB)
- **Table S5:** Multivariable analyses of predictors of Activities of Daily Living domain score (PDF, 1 page, 64 KB)
- **Table S6:** Multivariable analyses of predictors of Work-related Problems domain score (PDF, 1 page, 67 KB)
- **Table S7:** Multivariable analyses of predictors of Social Limitations domain score (PDF, 1 page, 66 KB)
- **Table S8:** Multivariable analyses of predictors of Intensity of Complaints domain score (PDF, 1 page, 6 KB)
- **Table S9:** Multivariable analyses of predictors of Emotional Complaints domain score (PDF, 1 page, 66 KB)
