# Supplementary material for: Quality of life 1 month after acute pulmonary embolism in emergency department patients
Source: Acad Emerg Med. Author manuscript; Available in PMC 2025 Apr 5. (PMC11971718; doi:10.1111/acem.14692)
Supplement: Table S2 [file NIHMS2065999-supplement-Table_S2.pdf]

Table S2: Characteristics of those completing PEmb-QoL versus those who did not*

	**PEmb-QoL completed (N = 788)**	**PEmb-QoL not completed (N = 913)**
**Age, years**		
Mean (SD)	58.9 (15.9)	60.0 (17.1)
Missing	1 (0.1%)	3 (0.3%)
**Sex**		
Female	405 (51.4%)	447 (49.0%)
Male	383 (48.6%)	466 (51.0%)
**Race**		
African American	220 (27.9%)	275 (30.1%)
Caucasian	527 (66.9%)	587 (64.3%)
Pacific Islander/Native Hawaiian	4 (0.5%)	1 (0.1%)
Asian	9 (1.1%)	8 (0.9%)
Native American	2 (0.3%)	9 (1.0%)
Unknown	21 (2.7%)	10 (1.1%)
Other	5 (0.6%)	23 (2.5%)
**Ethnicity**		
Hispanic	56 (7.1%)	62 (6.8%)
Not Hispanic	707 (89.7%)	827 (90.7%)
Unanswered	25 (3.2%)	23 (2.5%)
**Any cancer?**		
Yes	157 (19.9%)	261 (28.6%)
**BMI**		
Mean (SD)	32.6 (9.40)	30.1 (8.2)
**BMI > 30**		
Yes	422 (53.6%)	384 (42.8%)

**Systolic blood pressure, mmHg**		
Mean (SD)	134 (24.4)	131.6 (23.9)
**Heart rate, beats per minute**		
Mean (SD)	96.5 (21.4)	98.9 (21.4)
**Shock index**		
Mean (SD)	0.75 (0.240)	0.78 (0.25)
**Respiratory rate, breaths per minute**		
Mean (SD)	19.7 (3.94)	19.9 (4.9)
**Spontaneous breathing?**		
Yes	759 (96.3%)	876 (96.1%)
**O2 saturation**		
Mean (SD)	95.6 (4.11)	95.5 (4.84)
**Respiratory rate > 30 bpm**		
Yes	26 (3.3%)	60 (6.6%)
**Temperature, degrees Fahrenheit**		
Mean (SD)	98.2 (0.886)	98.2 (0.97)
**Transient hypotension prior to enrollment**		
Yes	53 (6.7%)	79 (8.7%)
**Preceding episodes of syncope**		
Yes	66 (8.4%)	89 (9.7%)
**Preceding episodes bradycardia? (< 40 bpm)**		
Yes	11.0 (1.4%)	14 (1.5%)
**GDE showing RVD?**		
No	532 (67.5%)	606 (68.4%)
Yes	236 (29.9%)	280 (31.6%)
Missing	20 (2.5%)	27 (3.0%)
**Prior diagnosis of PE or DVT**		
Yes	210 (26.6%)	216 (23.7%)

**Recent trauma**		
Yes	49.0 (6.2%)	76 (8.3%)
Missing	1 (0.1%)	1 (0.1%)
**Recent hospitalization?**		
Yes	244 (31.0%)	326 (35.7%)
**Clotting disorders (protein c, s, factor V...)**		
Yes	24 (3.0%)	26 (2.9%)
**Low risk sPESI?**		
Yes	315 (40.0%)	292 (32%)
**PE-SCORE points assigned**		
0	166 (21.1%)	165 (18.1%)
1 to 4 points	529 (67.1.%)	636 (69.6%)
5 to 10 points	48 (6.1%)	49 (5.4%)
Missing	45 (5.7%)	63 (6.9%)
**Low risk ESC?**		
No	694 (88.1%)	825 (90.4%)
Yes	94 (11.9%)	88 (9.6%)
**Natriuretic peptide elevation**		
Yes	309 (39.2%)	329 (37.3%)
Missing	39 (4.9%)	31 (3.4%)
**Troponin elevation**		
Yes	196 (24.9%)	242 (26.7%)
Missing	12 (1.5%)	8 (0.9%)
**Suspected/confirmed severe LV dysfunction?**		
Yes	32 (4.1%)	59 (7.6%)
Missing	2 (0.3%)	
**DVT ultrasound findings if assessed**	n= 471	n=547
No	137 (29.1%)	144 (26.3%)

Yes	334 (70.1%)	403 (73.7%)
**Acute clinical deterioration within 5 days?**		
No	632 (80.2%)	670 (73.4%)
Yes	156 (19.8%)	243 (26.6%)
**Reperfusion intervention within 5 days**		
Yes	44 (5.6%)	74 (8.1%)
**Anticoagulation compliance at 30 days**		
No	26 (3.3%)	31 (3.6%)
Yes	759 (96.3%)	499 (57.7%)
Missing	3 (0.4%)	48 (5.3%)
**Recurrence of VTE within 30 days**		
No	780 (99.0%)	807 (88.4%)
Yes	8 (1.0%)	14 (1.6%)
**Major bleeding within 30 days**		
Yes	21 (2.7%)	42 (4.7%)
**Family history of VTE**		
Yes	56 (7.1%)	59 (6.5%)
**Hormone replacement therapy**		
Yes	25 (3.2%)	31 (3.4%)
**Indwelling catheter**		
Yes	44 (5.6%)	68 (7.5%)
**Suspected/confirmed systemic infection**		
Yes	41.0 (5.2%)	48 (5.3%)
**Severe renal impairment? (creatinine > 2.0)**		
Yes	25 (3.2%)	43 (4.7%)
**Severe liver impairment?**		
Yes	8 (1.0%)	13 (1.4%)
**Chronic pulmonary disease**		

Yes	119 (15.1%)	127 (13.9%)
**AIDS (not just HIV positive)**		
Yes	3 (0.4%)	9 1.0%)
**Is CT RV/LV ratio 1.0 or more**		
Yes	265 (33.6%)	276 (30.8%)
Missing	17 (2.2%)	19 (2.1%)
**Anticoagulant initiated in ED?**		
Yes	739 (93.8%)	822/906 (90.7%)
Missing	2 (0.3%)	7 (0.8%)
**Active bleeding or high risk of bleeding including high risk post-operative state?**		
Yes	69 (8.8%)	108 (12.0%)
Missing	8 (1.0%)	11 (1.2%)
**Oxygen supply to maintain oxygen saturation > 90% for > 24 hours?**		
1	299 (37.9%)	384 (39.2%)
Missing	3 (0.4%)	11 (1.1%)
**GDE showing RVD?**		
No	532 (67.5%)	606 (68.4%)
Yes	236 (29.9%)	280 (31.6%)
Missing	20 (2.5%)	27 (3.0%)
**Reperfusion intervention within 5 days**		
Yes	44.0 (5.6%)	74 (8.1%)
**Anticoagulation compliance at 30 days**		
No	26 (3.3%)	31 (3.6%)
Yes	759 (96.3%)	499 (57.7%)
Missing	3 (0.4%)	48 (5.3%)
**Prior diagnosis of PE or DVT**		
Yes	210 (26.6%)	216 (23.7%)

**Recent trauma**		
Yes	49.0 (6.2%)	76 (8.3%)
Missing	1 (0.1%)	1 (0.1%)
**Recent hospitalization?**		
Yes	244 (31.0%)	326 (35.7%)
**Clotting disorders (protein c, s, factor V...)**		
Yes	24 (3.0%)	26 (2.9%)
Missing	1 (0.1%)	3 (0.3%)
**Pulmonary embolism diagnosed during anticoagulation treatment or with INR > 1.7?**		
Yes	55 (7.0%)	57 (6.3%)
**Intractable pain?**		
Yes	22.0 (2.8%)	32 (3.6%)
Missing	3 (0.4%)	15 (1.6%)
**Medical or social reason for treatment in the hospital > 24 hours?**		
Yes	392 (49.7%)	424 (47.1%)
Missing	5 (0.6%)	
**Hospital length of stay, hours**		
Mean (SD)	100 (118)	123.5 (147.3)
**Subsequent rehospitalization**		
Yes	87 (11.1%)	136 (15.2%)
Missing	1 (0.1%)	17 (1.9%)

* Abbreviations: PEmb-QoL = pulmonary embolism quality of life questionnaire, GDE = goal directed echocardiography, RVD = right ventricular dysfunction, PE = pulmonary embolism, DVT = deep venous thromboembolism, sPESI = simplified pulmonary embolism severity index, PE-SCORE = pulmonary embolism short-term clinical outcomes risk estimation, ESC = European Society of Cardiology, LV = left ventricle, VTE = venous thromboembolism, BMI =

body mass index, AIDS = acquired immunodeficiency syndrome, HIV = human immunodeficiency virus, CT = computed tomography, ED = emergency department.
