# Supplementary material for: Quality of life 1 month after acute pulmonary embolism in emergency department patients
Source: Acad Emerg Med. Author manuscript; Available in PMC 2025 Apr 5. (PMC11971718; doi:10.1111/acem.14692)
Supplement: Table S3 [file NIHMS2065999-supplement-Table_S3.pdf]

Table S3: Results of 2 non-domain pulmonary embolism quality of life (PEmb-QoL) questions

PEmb-QoL questions	No subsequent rehospitalization (N = 700)	Subsequent rehospitalization (N = 87)	Overall (N = 788)
Q2: At what time of the day are your symptoms most intense?			
On waking up	105 (15.2%)	14 (16.2%)	119 (15.5%)
Mid-day	369 (5.2%)	3 (3.5%)	39 (5.0%)
End of day	33 (4.8%)	2 (2.3%)	35 (4.5%)
At night	58 (8.4%)	14 (16.3%)	72 (9.0%)
Any time of the day	222 (32.1%)	26 (30.2%)	248 (32%)
Never	244 (35.3%)	27 (31.4%)	272 (35%)
Missing	8 (1.1%)	1(1.1%)	9 (1.1%)
Q3: Compared to one year ago, how would you rate the condition of your lungs in general now?			
Much better now	36 (5.2%)	6 (6.9%)	42 (5.4%)
Somewhat better	41(5.9%)]	4(4.6%)	45 (5.7%)
About the same	136 (19.5%)	14 (16.1%)	150 (19.1%)
Somewhat worse now	199 (28.6%)	17 (19.5%)	216 (27.6%)
Much worse now	98 (14.1%)	18 (20.7%)	116 (14.8%)

I did not have lung problems before	186 (26.7%)	28 (32.2%)	215 (27.4%)
Missing	0	0	0
