# Supplementary material for: Quality of life 1 month after acute pulmonary embolism in emergency department patients
Source: Acad Emerg Med. Author manuscript; Available in PMC 2025 Apr 5. (PMC11971718; doi:10.1111/acem.14692)
Supplement: Table S4 [file NIHMS2065999-supplement-Table_S4.pdf]

Table S4: Multivariable analyses of predictors of Frequency of Complaints domain score*

	Frequency of Complaints (transformed score on 100-point scale)		
Predictors	*Estimates*	*Confidence Interval*	*P-value*
(Intercept)	15.33	12.86–17.80	**<0.001**
PE-SCORE points	-0.93	-2.24–0.38	0.163
Clinical deterioration event	2.35	-1.73–6.42	0.259
RVD plus reperfusion intervention	-4.03	-11.71–3.65	0.303
RVD without reperfusion intervention	-0.77	-4.97–3.42	0.718
Subsequent rehospitalization	9.63	5.35–13.91	**<0.001**
Hospital length of stay	0.00	-0.02–0.03	0.827
Observations	788		
R2 / R2 adjusted	0.034 / 0.027		

* Abbreviations: PE-SCORE = pulmonary embolism short-term clinical outcomes risk estimation, RVD = right ventricular dysfunction
