# Supplementary material for: Quality of life 1 month after acute pulmonary embolism in emergency department patients
Source: Acad Emerg Med. Author manuscript; Available in PMC 2025 Apr 5. (PMC11971718; doi:10.1111/acem.14692)
Supplement: Table S5 [file NIHMS2065999-supplement-Table_S5.pdf]

Table S5: Multivariable analyses of predictors of Activities of Daily Living domain score

	**Activities of Daily Living (transformed score on 100 point scale)**		
Predictors	*Estimates*	*Confidence Interval*	*P-value*
(Intercept)	26.36	22.78 – 29.93	**<0.001**
PE-SCORE points	-1.09	-2.52 – 0.35	0.138
Clinical deterioration event	-1.34	-6.81 – 4.14	0.632
Subsequent rehospitalization	14.00	7.79 – 20.22	**<0.001**
Hospital length of stay	0.13	0.09 – 0.17	**<0.001**
Observations	788		
R2 / R2 adjusted	0.087 / 0.082		

* Abbreviations: PE-SCORE = pulmonary embolism short-term clinical outcomes risk estimation, RVD = right ventricular dysfunction
