# Supplementary material for: Quality of life 1 month after acute pulmonary embolism in emergency department patients
Source: Acad Emerg Med. Author manuscript; Available in PMC 2025 Apr 5. (PMC11971718; doi:10.1111/acem.14692)
Supplement: Table S6 [file NIHMS2065999-supplement-Table_S6.pdf]

Table S6: Multivariable analyses of predictors of Work-related Problems domain score

	Work-related Problems (transformed score on 100-point scale)		
Predictors	*Estimates*	*Confidence Interval*	*P-value*
(Intercept)	44.70	38.96–50.45	**<0.001**
PE-SCORE points	0.71	-2.33–3.75	0.647
Clinical deterioration event	-0.15	-9.62–9.32	0.975
RVD plus reperfusion intervention	-8.93	-26.78–8.91	0.326
RVD without reperfusion intervention	-6.24	-15.99–3.51	0.209
Subsequent rehospitalization	18.48	8.54–28.43	**<0.001**
Hospital length of stay	0.12	0.06–0.18	**<0.001**
Observations	788		
R2 / R2 adjusted	0.043 / 0.036		

* Abbreviations: PE-SCORE = pulmonary embolism short-term clinical outcomes risk estimation, RVD = right ventricular dysfunction
