# Supplementary material for: Quality of life 1 month after acute pulmonary embolism in emergency department patients
Source: Acad Emerg Med. Author manuscript; Available in PMC 2025 Apr 5. (PMC11971718; doi:10.1111/acem.14692)
Supplement: Table S7 [file NIHMS2065999-supplement-Table_S7.pdf]

Table S7: Multivariable analyses of predictors of Social Limitations domain score

	Social Limitations (transformed score on 100 point scale)		
Predictors	*Estimates*	*Confidence Interval*	*p value*
(Intercept)	18.52	14.39–22.64	**<0.001**
PE-SCORE points	0.27	-1.91–2.46	0.807
Clinical deterioration event	1.12	-5.68–7.91	0.747
RVD plus reperfusion intervention	-2.77	-15.58–10.03	0.671
RVD without reperfusion intervention	-5.00	-11.99–2.00	0.161
Subsequent rehospitalization	13.34	6.20–20.47	**<0.001**
Hospital length of stay	0.10	0.06–0.15	**<0.001**
Observations	788		
R2 / R2 adjusted	0.057 / 0.050		

* Abbreviations: PE-SCORE = pulmonary embolism short-term clinical outcomes risk estimation, RVD = right ventricular dysfunction
