# Supplementary material for: Quality of life 1 month after acute pulmonary embolism in emergency department patients
Source: Acad Emerg Med. Author manuscript; Available in PMC 2025 Apr 5. (PMC11971718; doi:10.1111/acem.14692)
Supplement: Table S8 [file NIHMS2065999-supplement-Table_S8.pdf]

Table S8: Multivariable analyses of predictors of Intensity of Complaints domain score

	Intensity of Complaints (transformed score on 100- point scale)		
Predictors	*Estimates*	*Confidence Interval*	*P-value*
(Intercept)	20.79	17.59–23.99	**<0.001**
PE-SCORE points	-0.10	-1.80–1.60	0.908
Clinical deterioration event	1.47	-3.80–6.75	0.583
RVD plus reperfusion intervention	-6.63	-16.57–3.31	0.191
RVD without reperfusion intervention	-4.80	-10.23–0.63	0.083
Subsequent rehospitalization	12.13	6.59–17.67	**<0.001**
Hospital length of stay	0.01	-0.02–0.05	0.450
Observations	788		
R2 / R2 adjusted	0.033 / 0.026		

* Abbreviations: PE-SCORE = pulmonary embolism short-term clinical outcomes risk estimation, RVD = right ventricular dysfunction
