# Supplementary material for: Quality of life 1 month after acute pulmonary embolism in emergency department patients
Source: Acad Emerg Med. Author manuscript; Available in PMC 2025 Apr 5. (PMC11971718; doi:10.1111/acem.14692)
Supplement: Table S9 [file NIHMS2065999-supplement-Table_S9.pdf]

Table S9: Multivariable analyses of predictors of Emotional Complaints domain score

	**Emotional Complaints (transformed score on 100 point scale)**		
Predictors	*Estimates*	*Confidence Interval*	*p value*
(Intercept)	17.88	15.32–20.44	**<0.001**
PE-SCORE points	-0.10	-1.45–1.25	0.885
Clinical deterioration event	1.48	-2.73–5.70	0.490
RVD plus reperfusion intervention	-3.69	-11.63–4.25	0.362
RVD without reperfusion intervention	-1.14	-5.48–3.20	0.606
Subsequent rehospitalization	7.71	3.29–12.14	**0.001**
Hospital length of stay	0.01	-0.01–0.04	0.361
Observations	788		
R^2 / R^2 adjusted	0.019 / 0.011		

* Abbreviations: PE-SCORE = pulmonary embolism short-term clinical outcomes risk estimation, RVD = right ventricular dysfunction
